# Immune Dysfunction Associated with Abnormal Bone Marrow-Derived Mesenchymal Stroma Cells in Senescence Accelerated Mice

**DOI:** 10.3390/ijms17020183

**Published:** 2016-01-29

**Authors:** Ming Li, Kequan Guo, Yasushi Adachi, Susumu Ikehara

**Affiliations:** 1 Department of Stem Cell Disorders, Kansai Medical University, Hirakata City, Osaka 573-1010, Japan; liming@hirakata.kmu.ac.jp (M.L.); adachiya250@gmail.com (Y.A.); 2 Department of Cardiac Surgery, Beijing Institute of Heart, Lung and Blood Vessel Disease, Beijing Anzhen Hospital Affiliated to Capital Medical University, Beijing 100029, China; guokequan@hotmail.com; 3 Division of Surgical Pathology, Toyooka Hospital, Hyogo 668-8501, Japan

**Keywords:** SAMP10, bone marrow-derived MSCs, cell cycle, PI3K, MAPK

## Abstract

Senescence accelerated mice (SAM) are a group of mice that show aging-related diseases, and SAM prone 10 (SAMP10) show spontaneous brain atrophy and defects in learning and memory. Our previous report showed that the thymus and the percentage of T lymphocytes are abnormal in the SAMP10, but it was unclear whether the bone marrow-derived mesenchymal stroma cells (BMMSCs) were abnormal, and whether they played an important role in regenerative medicine. We thus compared BMMSCs from SAMP10 and their control, SAM-resistant (SAMR1), in terms of cell cycle, oxidative stress, and the expression of PI3K and mitogen-activated protein kinase (MAPK). Our cell cycle analysis showed that cell cycle arrest occurred in the G0/G1 phase in the SAMP10. We also found increased reactive oxygen stress and decreased PI3K and MAPK on the BMMSCs. These results suggested the BMMSCs were abnormal in SAMP10, and that this might be related to the immune system dysfunction in these mice.

## 1. Introduction

The immune system has been studied in neurological diseases, with neurons and microglial cells being found to be regulated by immune molecules [1]. Hematopoietic stem cell (HSC) proliferation is supported by mesenchymal stem cells in the bone marrow, and HSCs may differentiate into myeloid and lymphoid progenitors. The percentage of myeloid cells is higher than that of lymphoid cells in the bone marrow of old subjects [2]. Mesenchymal stem cells have the capacity for self-renewal and differentiation into osteoblasts, chondrocytes and adipocytes. Moreover, mesenchymal stem cells also secrete factors that help to prevent immune reactions [3]. However, oxidative stress appears to reduce the number and function of adult stem cells in the mesenchymal stem cells [4,5]. Additionally, one report has shown that bone marrow-derived mesenchymal stem cells differentiate into adipocytes that negatively regulate hematopoiesis [6].

The senescence-accelerated mouse prone 10 (SAMP10) is a mouse model for neurodegenerative diseases. This strain shows age-related brain atrophy and deficits in learning and memory [7]. A previous report of ours showed that thymic epithelial cells and T lymphocytes were dysfunctional in SAMP10, but that there was an improvement after the transplantation of normal bone marrow stem cells [8]. However, it was unclear whether bone marrow-derived mesenchymal stroma cells (BMMSCs) were abnormal in SAMP10. We thus compared the BMMSCs of SAMP10 and SAMR1, with the results showing that cultured BMMSCs were abnormal in SAMP10 compared with SAMR1, suggesting that BMMSCs might be associated with the immune dysfunction in SAMP10.

## 2. Results and Discussion

### 2.1. Analysis of Immune Cells in Peripheral Blood

**Figure 1:**
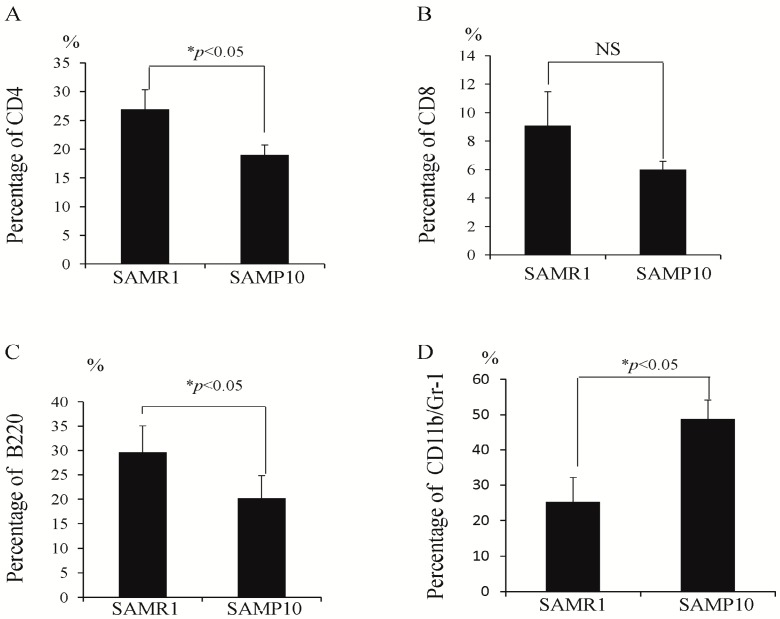
(**A**–**D**) Percentages of CD4, CD8, B220 and CD11b/Gr-1 in the peripheral blood of SAMP10 and SAMR1. The percentages of CD4-positive cells (**A**); CD8-positive cells (**B**); B220-positive cells (**C**); CD11b/Gr-1-positive cells (**D**).

The peripheral blood of SAMP10 and SAMR1 was analyzed by fluorescence activated cell sorting (FACS). As shown in Figure 1A,B, the percentages of CD4-positive cells were significantly lower in the SAMP10 than SAMR1 (19.0% ± 1.7% *vs.* 26.9% ± 3.4%, *p* < 0.05), while there was no significant difference in CD8-positive cells (6.0% ± 0.6% *vs.* 9.1% ± 2.4%, non-significant (NS)). Figure 1C,D show that the percentages of B220-positive cells were significantly lower, and CD11b/Gr-1 double-positive cells were significantly higher (20.2% ± 4.6% *vs.* 29.6% ± 5.5%, *p* < 0.05; 48.7% ± 5.4% *vs.* 25.3% ± 6.9%, *p* < 0.05) in the SAMP10 than SAMR1. Total numbers of lymphoctes in SAMP10 and SAMR1 were (4.5 ± 0.6) × 10^5^ and (6.6 ± 1.1) × 10^5^, *p* < 0.05. The percentages of CD4^+^CD8^+^ and CD4^−^CD8^−^ in the thymus of SAMP10 and SAMR1 were 72% ± 3.1% *vs.* 81% ± 2.8%, *p* < 0.05 and 14.0% ± 2.1% *vs.* 9.6% ± 1%. *p* < 0.05.

### 2.2. Analyses of Adiponectin and Cytokines

**Figure 2:**
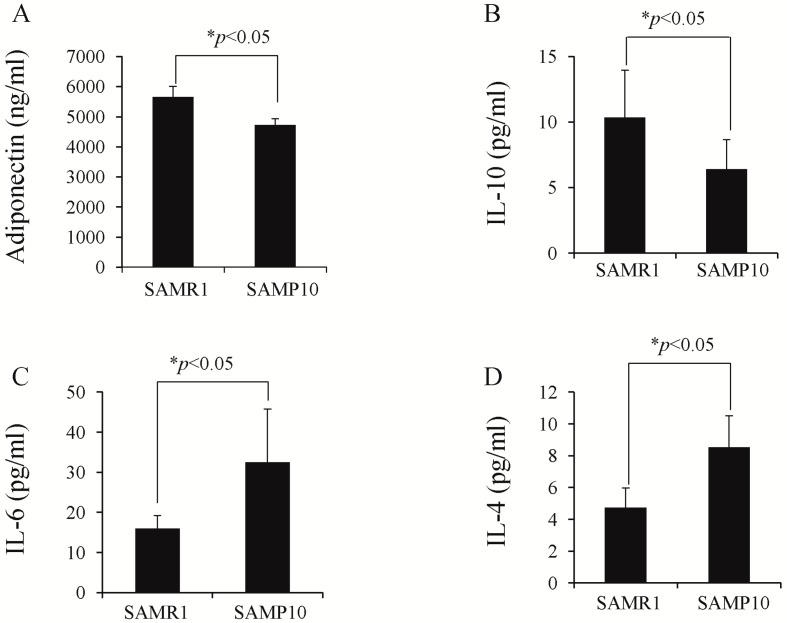
(**A**–**D**) Comparison of plasma levels of adiponectin and cytokines in the SAMP10 and SAMR1. The plasma levels of adiponectin in peripheral blood; (**B**–**D**) The plasma levels of IL-10 (**B**), IL-6 (**C**) and IL-4 (**D**).

Adiponectin is an adipokine produced by adipocytes. Figure 2A shows that the plasma level of adiponectin was significantly lower in the SAMP10 than in the SAMR1 (4725.3 ± 207.5 *vs.* 5651.0 ± 349.2 ng/mL, *p* < 0.05). Similarly, there was a significant difference in body weight (37 ± 2.8 *vs.* 32.2 ± 3.5 g, *p* < 0.05). Figure 2B shows that the plasma level of IL-10 was significantly lower in the SAMP10 than SAMR1 (6.4 ± 2.3 *vs.* 10.3 ± 3.6 pg/mL, *p* < 0.05). In contrast, plasma IL-4 and IL-6 levels were significantly higher in the SAMP10 than SAMR1 (32.5 ± 13 *vs.* 15.6 ± 3.2, 8.5% ± 1.9% *vs.* 4.7 ± 1.2 pg/mL) (Figure 2C,D).

### 2.3. Cell Cycle and Oxidative Stress Analysis in BMMSCs

**Figure 3:**
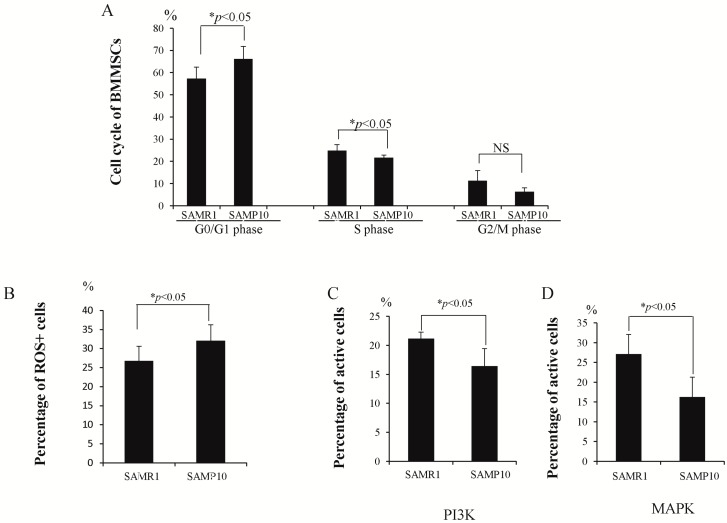
**A**–**D** Analysis of cell cycle, ROS and activities of PI3K and MAPK of BMMSCs between SAMP10 and SAMR1. (**A**) The percentage of BMMSCs in the G0/G1 phase was significantly higher and that in the S phase was significantly lower in SAMP10 than SAMR1. There was no significant difference in the G2/M phase between the two groups. The percentage of ROS-positive BMMSCs increased significantly in SAMP10 (**B**), while the active cells of PI3K and MAPK significantly decreased in the BMMSCs of SAMP10 but not SAMR1 (**C**,**D**). NS: non-significant.

The cell cycle of cultured BMMSCs was measured, Figure 3A showing that the percentage of BMMSCs was significantly higher in the SAMP10 than SAMR1 during the G0/G1 phase (66.1% ± 5.7% *vs.* 57.2% ± 5.3%, *p* < 0.05). In contrast, it was significantly lower in the SAMP10 than SAMR1 during the S phase (21.6% ± 1.3% *vs.* 24.9% ± 2.7%, *p* < 0.05). However, there was no significant difference during the G2/M phase (6.2% ± 1.8% *vs.* 11.2% ± 4.6%, *p* < 0.05). As shown in Figure 3B, the percentage of reactive oxygen stress (ROS)-positive-BMMSCs was significantly higher in the SAMP10 than SAMR1 (32.1% ± 4.1% *vs.* 26.7% ± 3.9%, *p* < 0.05). As shown in Figure 3C,D, PI3K and MAPK activities significantly decreased in the BMMSCs of SAMP10 (16.3% ± 3.1% *vs.* 21.1% ± 1.2%, 16.2% ± 4.9% *vs.* 27.0% ± 3.9%, *p* < 0.05) when compared with SAMR1.

### 2.4. Morphology of Bone Marrow and Brain 

**Figure 4:**
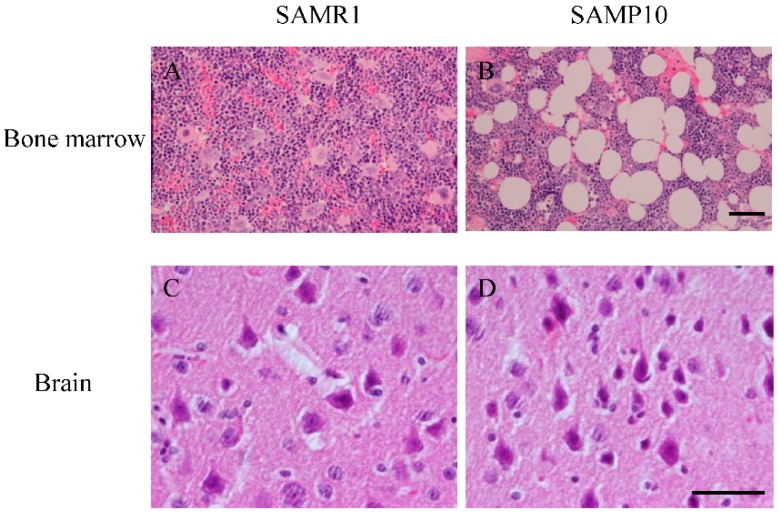
Morphology of bone marrow and brain in SAMP10 and SAMR1. (**A**,**B**) show hematoxylin and eosin (H&E) staining of the bone marrow. Many more adipocytes were found in the bone marrow of SAMP10 than SAMR1 mice. H&E staining showed no significant difference in the brain neurons (**C**,**D**). Scale bar = 25 μm.

Many more adipocytes were found in the bone marrow of SAMP10 (Figure 4B) than in the SAMR1 mice (Figure 4A). There was no significant difference in brain neurons between the two groups (Figure 4C,D).

### 2.5. Discussion

Learning ability and memory capacity both declined in SAMP10 [9]. These characteristic pathological phenotypes are similar to the age-associated disorders often observed in older people. The ninth-month-old and older SAMP10 mice failed to shorten the escape latency when these mice were tested for spatial learning using the Morris water maze [10]. Shimada’s group reported that SAMP10 showed brain aging and immunosenescence, and also that neuroinflammation is associated with age-related cognitive decline [11]. However, there are more inflammatory cytokines in SAMP10 less than 10-month-old. Our previous report also describes the age-related dysfunction of the immune system in SAMP10 from six-month-old SAMP10. Moreover, this immune dysfunction was improved by bone marrow stem cell replacement [8]. Bone marrow stem cells mainly include hematopoietic stem cells and BMMSCs. MSCs have been reported to modulate the immune response, and affect cellular differentiation and maturation [12]. However, because it was unclear whether the BMMSCs were abnormal in SAMP10, we elected to study eight-month-old SAMP10 to study the BMMSCs.

The immune system plays a role in modulating learning, memory and neural plasticity. When activated, the immune system upregulates the remodeling of neural circuits and promotes memory consolidation and neurogenesis [13]. BMMSCs not only support hematopoiesis, but also modulate immune functions. In our experiment, there were no significant differences in the number of fibroblast-colony forming units between the two groups (data not shown), suggesting that there was no significant difference in the number of BMMSCs between SAMP10 and SAMR1. However, analysis of the cell cycle showed that the percentage of BMMSCs in the G0/G1 phase was significantly higher in the SAMP10 than SAMR1, suggesting that cell cycle arrest occurred in the G0/G1 phase of SAMP10. Oxidative stress and DNA damage have been reported to induce cell senescence *in vitro* [14]. Moreover, the expression of reactive oxygen stress (ROS) levels was greater in the cultured BMMSCs of the SAMP10 than SAMR1, suggesting that oxidative stress, itself, was higher in the BMMSCs of the SAMP10. Angeles’s group reported that oxidative stress impairs regenerative capacity resulting from cell senescence in human MSCs [15]. BMMSCs from old mice showed decreased phosphor-protein kinase B(AKT) and more apoptosis under hypoxic conditions [16]. They also showed impaired proliferation and differentiation capacity, and decreased expression of AKT and Sirt1, but increased p53 and Bax, suggesting that the expression of Sirt1 was lower, while that of apoptotic and senescent genes was higher in the old mice. AKT/PI3K is an important intracellular signaling pathway. Both PI3K and MAPK play key roles in cell proliferation, differentiation and the stress response. Our results with the BMMSCs of SAMP10 showed that PI3K and MAPK activity decreased, suggesting that PI3K and MAPK were impaired in these mice.

In the peripheral blood, the percentages of Gr-1-positive cells increased and the percentages of CD4^+^ cells and B220^+^ cells decreased. Moreover, cytokines IL-6 and IL-4 increased and IL-10 decreased, suggesting that immune functions were impaired in SAMP10. One report has shown that aging increases the plasma levels of inflammatory cytokines, and that this was associated with dysregulated inflammation in the elderly [17]. Aging induced thymic atrophy, which induces impaired T cell development via the impaired maturation of thymocytes. Our results showed that the value of CD4^+^/CD8^+^ cells was higher in the SAMP10 than the controls. In contrast, there were fewer CD4^+^ or CD8^+^ cells in the peripheral blood in the SAMP10. Moreover, there were significant differences in the percentages of CD4^+^CD8^+^ and CD4^−^CD8^−^ in the thymus between the two groups, suggesting that the maturation of lymphocytes had been disturbed, possibly from thymic dysfunction. Further studies will focus on the relationship between the thymus and bone marrow stem cells in aged SAMP10. In our study, the decreased plasma adiponectin levels were detected in the SAMP10, but body weight was also reduced at the same time. Therefore, we could not conclude that the altered physiological function of the BMMSCs affected the exocrine properties of the adipocytes.

Neurofibrillary tangles and senile plaques are pathological changes seen in Alzheimer’s Disease, but no similar changes were found in the SAMP10 brain [18]. Moreover, Shimada’s group reported that pathological changes were observed in the brains of older SAMP10. In our studies, no neurofibrillary tangles or senile plaques were observed in the brains of SAMP10, and there was no significant difference in brain neuronal morphology between SAMP10 and SAMR1. In this series of experiments, we focused on comparing the differences between the BMMSCs of SAMP10 and SAMR1 to show that these differences may be associated with immunological dysfunctions in SAMP10.

The SAM strain, which includes SAMP and SAMR, was established as a novel murine model of senescence acceleration, the respective SAMP models having characteristic pathological phenotypes similar to those observed in elderly humans. SAMP10, a substrain of SAMP mice, show age-related deficits in memory, while SAMR1 shows no such deficits. We also have found an immune dysfunction in SAMP10, but not in SAMR1, and for this reason, our initial explorations focused on compared BMMSCs between in SAMP10 and SAMR1. However, the present study lacks a comparison between mice groups of different ages, and it is possible that the differences we observed between the SAMR1 and SAMP10 mice were due to the different genotypes rather than to different aging processes. Additional functional assays on the BMMSCs should therefore also be performed in the different age groups in order to fully understand the underlying mechanisms and the significance of the present findings. These issues will be addressed in future work, and we will also focus on the relationship between neuronal morphology and BMMSCs in SAMP10.

## 3. Materials and methods

### 3.1. Animals

Male SAMP10 and SAM resistant (SAMR1) mice were purchased from Shimizu Laboratory Supplies (Shizuoka, Japan). The Institutional Animal Care and Use Committee approved all procedures of the animal experiments at the Kansai Medical University (The project identification code 12-014, approved on 9 April 2012). Eight-month-old mice were used in the experiments. The same experiment was repeated three times (*n* = 7 in each group).

### 3.2. Flow Cytometric Analyses 

The peripheral blood was obtained from the tail vein of the mice, and the mononuclear cells isolated from the peripheral blood were stained with antibodies against FITC-CD4, FITC-CD8, PE-B220 and FITC-CD11b and PE-Gr-1 (BioLegend, San Diego, CA, USA) and analyzed by FACScan (BD Bioscience, San Jose, CA, USA) according to our previous report [9]. For thymus analysis, thymocytes were stained with antibodies against FITC-CD4 and PE-CD8, and then analyzed by FACScan. Isotype-matched immunoglobulins (BioLegend) were used as controls.

### 3.3. Immunochemistry

The bones and frontal cerebrum of SAMP10 and SAMR1 mice were removed and fixed in 10% formalin (Wako Pure Chemical Industries, Osaka, Japan) and then embedded in paraffin. The sections were stained with H&E. These sections were examined under a microscope.

### 3.4. BMMSC Culture

The bone marrow cells were collected from the femurs and tibias of SAMP10 and SAMR1 mice, and the BMMSCs were then cultured as described in our previous report [9]. Briefly, the BMMSCs were cultured using MesenCult media according to the manufacturer’s instructions (Stem Cell Technology, Vancouver, BC, Canada). After passage, the cells were further cultured in fresh αMEM (Life Technologies Corporation, NY, USA), including 10% FCS and 1% antibiotic-antimycotic solution (Life Technologies). The 3rd passage cells were used for analysis.

### 3.5. Measurement of Cell Cycle, Oxidative Stress and MAPK Pathway

The cell cycle was measured according to the manufacturer’s protocols in the Muse^TM^ cell cycle kit (EMD Millipore Corporation, Billerica, MA USA). It is important to obtain a single cell suspension prior to ethanol fixation. Briefly, each cell sample (1 × 10^6^/sample) was washed twice in phosphate buffered saltes (PBS), and then fixed for 3 h in 1 mL ice cold 70% ethanol at −20 °C. Each cell pellet (5 × 10^5^/sample) was then resuspended in 200 µL of Muse^TM^ cell cycle reagent after being washed twice, and then incubated for 30 min at room temperature, protected from light. The cell suspension sample was transferred to 1.5 mL micro centrifuge tube and analyzed on the Muse^®^ cell analyzer (Merck &Co. Inc., Darmstadt, Germany). Oxidative stress was measured according to the manufacturer’s protocol in the Muse^®^ oxidative stress kit (EMD Millipore Corporation). Briefly, 1 × 10^6^ cells/mL assay buffer were incubated with Muse^®^ oxidative stress working solution at 37 °C for 30 min and then mixed thoroughly before being analyzed on the Muse^TM^ cell analyzer (Millipore Corporation, Hayward, CA, USA).

PI3K and MAPK were measured by using Muse^TM^ PI3K activation dual detection kit and Muse^TM^ MAPK activation dual detection kit according to the manufacturer’s protocols for the kits. Briefly, 1 × 10^6^ cells in 500 µL assay buffer were fixed in fixation buffer, and the cells were then permeabilized by adding 1 mL ice-cold permeabilization buffer on ice for 5 min. The cells were stained by adding 5 µL of anti-Akt/PKB, PECy5 antibody or anti-ERK1/2, PECy5 antibody for 30 min in the dark at room temperature. The stained cells were suspended in 200 µL of assay buffer and analyzed using the Muse^TM^ cell analyzer.

### 3.6. Statistical Analysis

The results are represented as means ± SD. The Student’s *t* test was used to determine any statistical significance. A *p*-value of <0.05 was considered to be a significant difference.

## 4. Conclusions

In conclusion, this is the first report that BMMSC are abnormal in SAMP10, and that this might be associated with immune dysfunction. Future studies will focus on the relationship between BMMSCs and neurodegenerative diseases in SAMP10.
